# Oil- and Fuel-Resistant Rubber for Pressure Hoses Containing Carbon-Based Technological Waste as a Filler

**DOI:** 10.3390/polym18030330

**Published:** 2026-01-26

**Authors:** Abdirakym Nakyp, Elena Cherezova, Yulia Karaseva, Kanat Beknazarov, Rustam Tokpayev, Svetoslav Volfson, Mikhail Nauryzbayev

**Affiliations:** 1 Institute of Polymers, Kazan National Research Technological University, 68 K. Marx Str., 420015 Kazan, Russia; cherezova59@mail.ru (E.C.); karaseva_j@mail.ru (Y.K.); svolfson@kstu.ru (S.V.); 2 Center of Physico-Chemical Methods of Research and Analysis, Al-Farabi Kazakh National University, Al-Farabi Ave., 71, 050040 Almaty, Kazakhstan; kanat.beknazarov@kaznu.edu.kz (K.B.); rustamtokpaev@mail.ru (R.T.); nauryzbaev@cfhma.kz (M.N.)

**Keywords:** shungite, shungite concentrate, elastomer composite, physical-mechanical properties, thermal stability, oil- and fuel-resistance

## Abstract

Carbon-enriched concentrates based on shungite ore from rare-metal mining waste were obtained, and their effect on the properties of oil- and fuel-resistant carbon-black-filled rubber used for the production of pressure hoses was investigated. The shungite concentrates were produced by flotation followed by acid activation. A blend of nitrile butadiene rubber and butadiene–α-methylstyrene rubber was used as the elastomeric base. Carbon black was partially replaced with shungite fillers (5–15 phr). The presence of shungite was found to prolong both the scorch time and the optimum cure time of the rubber compounds, likely due to oxide impurities that interfere with the vulcanization activation process. Replacing carbon black with shungite ore and its flotation concentrate in the rubber formulations resulted in a decrease in Mooney viscosity compared to the samples without shungite fillers. Acid-activated shungite concentrate at contents above 5 phr increases the viscosity of the rubber compound. It was found that acid-activated shungite concentrate provides high tensile strength and excellent thermo-oxidative stability of the rubber, whereas the use of shungite ore above 5 phr reduces the tensile strength and causes significant changes in tensile properties upon thermo-oxidation. When exposed in oil, rubbers containing shungite fillers retain their mechanical properties, with the best resistance in hydrocarbon media observed for the rubber filled with acid-activated shungite concentrate.

## 1. Introduction

Dispersed fillers are widely used to enhance the performance of polymer composites, enabling the adjustment of their mechanical, dielectric, and thermal properties [1,2]. Carbon black remains the most widely used filler [3]; however, its capabilities are largely exhausted [4]. Moreover, the use of carbon black to achieve the desired performance properties is limited by environmental concerns associated with its production [3]. Consequently, there is active research into alternative dispersed fillers, including naturally occurring ones [5,6,7,8,9,10,11,12]. Carbon-containing minerals, particularly shungite, are considered promising candidates [13]. Incorporating such fillers can reduce the cost of rubbers while maintaining or even improving their performance properties [14,15,16,17,18,19].

Shungite is a mineral rock composed of amorphous carbon and silicate minerals [20,21,22]. It is characterized by a unique nanostructure with fullerene-like formations [23,24,25], which confer high chemical and adsorption activity. Reference [26] demonstrated that the addition of 10–20 parts per hundred rubber (phr) of shungite to a nitrile butadiene rubber formulation increases tensile strength and elongation at break. Partial replacement of carbon black with shungite filler also improves the processing properties of the rubber compound, as well as the frost resistance and thermal stability of the vulcanizates [27,28]. Study [29] showed that shungite imparts light-protective and bactericidal properties to polymer composites.

Nevertheless, practical use of shungite is associated with challenges, including considerable variations in carbon content, a high fraction of mineral impurities, and difficulties in achieving uniform particle dispersion within the polymer matrix [30,31]. Previous studies [26,32] have highlighted the role of shungite composition and demonstrated that carbon enrichment positively affects its effectiveness in rubber applications [33]. Physical and chemical methods are used to enrich natural shungite with carbon and remove mineral impurities [34,35]. In particular, flotation increases the carbon content and specific surface area of the filler [36,37], while alkali and acid activation reduces the amount of mineral impurities, including pyrite, and enhances the filler’s adhesion properties [38].

The analysis of changes in the aforementioned properties associated with the use of shungite-based fillers contributes to the valorization of technogenic waste and enables the determination of the optimal filler type and loading level.

The aim of this study is to investigate the effect of shungite ore from waste dumps of rare-metal mining (the Bakyrchik deposit, Eastern Kazakhstan, Kyzyl zone), its carbon-enriched concentrate obtained by flotation, and an acid-activated shungite concentrate on the physical-mechanical properties and thermal stability of oil- and fuel-resistant rubber. The scientific novelty of this work lies in substantiating the feasibility of using technogenic shungite waste, purposefully modified by flotation and acid activation, to increase the carbon fraction of the filler and reduce its mineral fraction. This approach enables controlled modification of the vulcanization characteristics of rubber compounds, the physical-mechanical properties, and the thermo-oxidative stability of oil- and fuel-resistant elastomeric composites when carbon black is partially replaced.

## 2. Materials and Methods

### 2.1. Materials

A polymer matrix consisting of nitrile butadiene rubbers of BNKS-18 AMN and BNKS-28 AMN grades (Technical Conditions TU 38.30313-2006, 2nd group, Krasnoyarsk Synthetic Rubber Plant JSC, Krasnoyarsk, Russia) and butadiene–α-methylstyrene rubber of SBR-1705 HI-AR grade (grade 1, group 1, State Standard GOST 11138-2019 [39], Omsk Rubber Plant JSC, Omsk, Russia) was used. Technical sulfur (grade 9995, 1st class, SERA CJSC, Orenburg, Russia) was used as a vulcanizing agent. Benzothiazole disulfide (MBTS, 2,2′-dithiobis(benzothiazole), State Standard GOST 7087-75 [40], BINA Group LLC, Moscow, Russia) served as vulcanization accelerator. Zinc oxide (ZnO, grade A, Empils-Zinc LLC, Rostov-on-Don, Russia) and stearic acid (grade T-32, Nefis Cosmetics JSC, Kazan, Russia) were used as vulcanization activators. The rubber compound also included the antioxidant N-phenyl-N′-isopropyl-p-phenylenediamine (IPPD) (State Standard GOST 5234-78 [41], Neftekhim Innovations LLC, Nizhny Novgorod, Russia). Dibutyl phthalate (DBP) (State Standard GOST 8728-88 [42], purity 99.5%, EcoTEK LLC, Moscow, Russia), a petroleum resin (Technical Conditions TU 2451-01-51513617-2007, Sibplast grade, EcoTEK LLC, Moscow, Russia), and petroleum bitumen (State Standard GOST 6617-2021 [43], BN 90/10 grade, LUKOIL-Volgogradneftepererabotka LLC, Volgograd, Russia) were used as softeners (plasticizers). The filler was carbon black of grades P 803 (Technical Conditions TU 20.13.21-001-04639704-2017, ash content not more than 2%, specific surface area 14–18 m^2^/g, EcoPolza PCF LLC, Astrakhan, Russia) and P 514 (Technical Conditions TU 20.13.21-001-04639704-2017, external specific surface area 42 ± 15 m^2^/g, EcoPolza PCF LLC, Astrakhan, Russia).

In the rubber compound, 5–15 phr of carbon black grade P 803 was partially replaced with a shungite filler derived from waste dumps of rare-metal mining (the Bakyrchik deposit, Kazakhstan), with its carbon-enriched concentrate obtained by flotation, and with an acid-activated shungite concentrate.

### 2.2. Preparation of Shungite Fillers

Preliminary preparation of the shungite ore (ShO) was carried out by crushing it in a grinding unit to obtain particles with sizes of 1–20 μm. Carbon enrichment of the shungite ore was performed by flotation according to the procedure described in [24,30], using a laboratory flotation machine FML-3 (Research and Engineering Corporation “Mekhanobr-Tekhnika”, St. Petersburg, Russia) with a cell volume of 3.0 L, under the selected reagent regime at a pulp temperature of 15 ± 1 °C. During flotation enrichment of ShO, pine oil Flotol 5219 (frother, 1260 g/t) and kerosene TS-1 (collector, 1134 g/t) were used; these reagents interact selectively with the minerals constituting the shungite ore. The flotation time was 10 min. After completion of the process, the suspension was filtered, and the shungite concentrate (ShC) was dried in a drying oven at 105 ± 5 °C to constant mass.

Acid activation of the shungite concentrate was carried out in a glass reactor using a 10% aqueous solution of hydrochloric acid according to the procedure described in [44,45]. The process was conducted at 50 °C under stirring for 6 h using a magnetic stirrer (300 rpm). Upon completion of the activation, the suspension was filtered. The precipitate on the filter was washed with demineralized water to a neutral pH value. After neutralization, the precipitate of the acid-activated shungite concentrate (ShC(a)) was dried in a drying oven at 105 ± 5 °C to constant mass. The particle size after modification remained in the range of 1–20 µm.

### 2.3. Preparation of Rubber Compounds and Vulcanizates

The rubber compounds were prepared in a closed laboratory internal mixer, a Brabender Plasti-Corder^®^ Lab-Station W50 E (Brabender, Duisburg, Germany), for 9 min at a temperature of 60 ± 3 °C and a rotor speed of 60 rpm. The mixing chamber volume was 50 cm^3^, with a fill volume of 85%. An industrially recommended formulation for pressure hoses (Sample C, Table 1) was used as the base recipe. In the experimental samples, part of carbon black (CB) grade P 803 was replaced with a filler based on shungite ore. CB P 803 was partially substituted with the shungite filler in the amount of 5–15 phr, in steps of 5 phr. The loading levels of shungite filler were selected based on an analysis of the literature data [17,24]. The vulcanizing agent (sulfur) was introduced during mixing in the internal mixer at the final stage, 1.0 min before the end of the mixing process.

Vulcanization of the rubber compounds was carried out in a laboratory vulcanization press with induction-heated plates, model 100-400-2E (Krasin Plant JSC, Kirov, Russia), at 160 °C in accordance with State Standard GOST 269-66. The thickness of the test sheets was 2.0 ± 0.2 mm.

### 2.4. Methods for the Characterization of Shungite Fillers, Rubber Compounds, and Vulcanizates

The microstructure and energy-dispersive analysis of the samples were carried out using a SEM3200 scanning electron microscope (CIQTEK Co., Ltd., Anhui, Hefei, China) equipped with an energy-dispersive X-ray spectroscopy (EDS) system with a tungsten cathode (XFlash Detector 730M-300, Bruker, Billerica, MA, USA), which enabled additional determination of the chemical composition of the examined regions. The investigations were performed at an accelerating voltage of 15 kV in low-vacuum mode. Backscattered electron (BSE) detectors were used to obtain the micrographs.

The elemental composition of the shungite fillers was examined using a 1430 VP scanning electron microscope (LEO Electron Microscopy Ltd., Oberkochen, Germany) coupled with an energy-dispersive X-ray spectrometer Quantax 200 (Bruker AXS, Karlsruhe, Germany).

The specific surface area of the shungite fillers was determined by the Brunauer–Emmett–Teller (BET) method using thermodesorption of an adsorbed gas on a “Sorbtometr” analyzer (Katakon JSC, Cheboksary, Russia) with liquid nitrogen as the coolant.

The thermal stability of the shungite fillers was evaluated in dynamic mode in the temperature range of 25–800 °C, and the influence of the shungite fillers on the thermal stability of the vulcanizates was assessed in the range of 25–600 °C in a nitrogen atmosphere using a STA 6000 thermal analyzer (PerkinElmer, Waltham, MA, USA) at a heating rate of 5 °C/min.

Dynamic mechanical parameters (tan δ) of the vulcanizates were studied using a DMA 242 C dynamic mechanical analyzer (NETZSCH Group, Selb, Germany) in the temperature range from −65 to +65 °C at a heating rate of 5 °C/min and a frequency of 1 Hz, in penetration mode.

Fourier-transform infrared (FTIR) spectra of the shungite fillers were recorded using a Nicolet iS10 FTIR spectrometer (Thermo Fisher Scientific, Waltham, MA, USA). The measurements were carried out in the range 600–4000 cm^−1^ with a spectral resolution of 2 cm^−1^ in ATR mode.

The rheometric characteristics of the rubber compounds were determined using an MDR GT-3000-A moving-die rheometer (GOTECH, Taichung City, Taiwan) at 160 °C for 30 min in accordance with State Standard GOST 34751-2021 [46] (ASTM D5289 [47]).

Mooney viscosity of the rubber compounds was determined using a Mooney viscometer UGT 7080S2 (GOTECH, Taichung City, Taiwan) in accordance with ASTM D1646 [48]. The measurements were carried out at 100 °C, with a 1 min preheating period followed by a 4 min test (ML (1 + 4) 100 °C).

The physical-mechanical properties of the rubber samples were evaluated according to State Standard GOST 270-75 [49] by tensile strength, elongation at break which were determined on the universal electromechanical testing machine TRM-P 50 C1 Tochline (NPO Tochpribor LLC, Rostov-on-Don, Russia). Shore A hardness of the samples was measured on a portable hardness tester TH-200 (Time Group, Beijing, China) according to State Standard GOST 263-75 [50]. Rebound resilience was determined on a Shoba type rebound resilience tester UMR-1 (Polymermash Group LLC, St. Petersburg, Russia) according to State Standard GOST 27110-86 [51].

Stability of the rubber properties to thermal aging in air was evaluated from the changes in the physicomechanical characteristics (tensile strength, elongation at break, rebound resilience and Shore A hardness) after conditioning at 100 ± 2 °C for 72 h. The tests were carried out in accordance with State Standard GOST 9.024-74 [52].

The resistance of the elastomeric compositions to the action of hydrocarbons was assessed from the change in tensile strength after exposure in oil for 3 days at 23 ± 2 °C. These tests were also performed in accordance with State Standard GOST 9.024-74. The composition of the oil is given in Table 2.

## 3. Results and Discussion

Shungite from the Bakyrchik deposit is classified as anthraxolite (a solid bitumen system) and is characterized by turbostratic (disordered) stacking of layers and a high content of mineral impurities [36,53,54]. In this study, the shungite ore was subjected to preliminary preparation aimed at carbon enrichment. Beneficiation of the pre-ground shungite ore was carried out by flotation, followed by acid activation of the resulting shungite concentrate.

**Figure 1:**
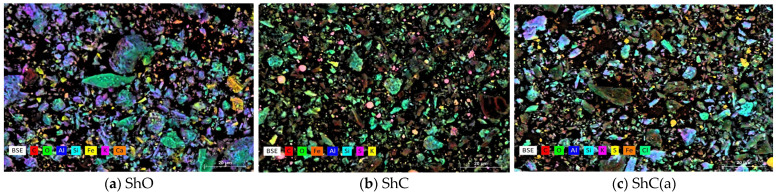
SEM images of the shungite fillers. (**a**) ShO [55]; (**b**) ShC; (**c**) ShC(a).

Figure 1 shows mapping images of the shungite fillers obtained by scanning electron microscopy (SEM). The samples represent solid dispersed systems with different microphase contents. The elemental composition and its quantitative distribution in the samples were determined by energy-dispersive X-ray spectroscopy.

The morphology and chemical composition of the shungite fillers differ markedly. The SEM images (Figure 1) indicate that the particles of shungite ore (ShO), concentrate (ShC), and acid-activated concentrate (ShC(a)) have an anisotropic shape and a broad size distribution, typical of coarsely ground powders.

The elemental composition (Table 3) indicates a significant carbon enrichment of the concentrate and acid-activated concentrate. The carbon content in the initial shungite ore (ShO) is 11%, whereas in the shungite concentrate (ShC) it reaches 39%, and in the acid-activated concentrate (ShC(a)) 55%. At the same time, beneficiation markedly reduces the mineral fraction. For example, the SiO_2_ content decreases from 49% in ShO, to 13% in ShC, and to 9% in ShC(a) (Table 3). Acid activation of ShC leads to partial dissolution of the silicate phase, which is accompanied by an increase in the specific surface area of the filler from 9 m^2^/g (ShO) to 17 m^2^/g (ShC) and 23 m^2^/g (ShC(a)) (Table 3). The increase in specific surface area can be attributed both to the removal of mineral components and to the disaggregation of filler particles, which enhances the reinforcing effect through physical factors. At the same time, acid treatment enriches the filler surface with the carbonaceous phase and increases the proportion of oxygen-containing functional groups, thereby altering the chemical nature of the filler surface. The combination of these factors—an increased geometric contact area and modification of surface chemistry—creates favorable conditions for enhanced physicochemical interactions between the shungite filler and the rubber matrix and explains the observed increase in the reinforcing effect in compositions containing ShC(a).

**Figure 2:**
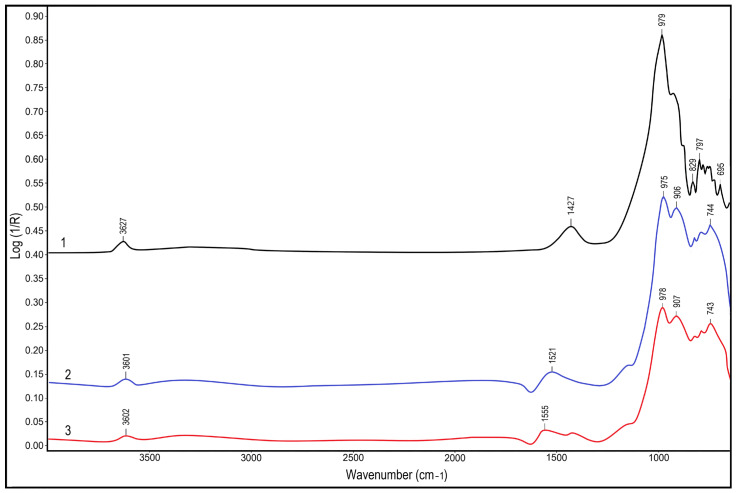
IR spectra of the shungite fillers: 1—ShO, 2—ShC, 3—ShC(a).

Figure 2 presents the IR spectra of the initial shungite ore, the shungite concentrate, and the acid-activated shungite concentrate. In the spectrum of the initial material, an intense band with a maximum at 3626 cm^−1^ is observed, corresponding to the stretching vibrations of OH groups of silicate minerals, along with a broad absorption region in the range of 1427–900 cm^−1^ associated with Si–O and Al–O bond vibrations.

Transition to the shungite concentrate is accompanied by a shift in the OH stretching band to 3617 cm^−1^ and the appearance of bands at 1859 and 1521 cm^−1^, indicating an increased contribution of the carbonaceous phase and oxygen-containing functional groups. After acid treatment, the intensity of bands characteristic of the silicate component (1650–900 cm^−1^) decreases markedly, while the band near 1883 cm^−1^, attributed to carbonyl (C=O) groups, becomes more pronounced.

The obtained IR spectral data indicate a reduction in the mineral phase content and the development of a functionalized carbonaceous surface of the filler as a result of acid activation. These changes may promote enhanced physicochemical interactions between the filler and the rubber matrix as well as components of the vulcanization system; however, they do not constitute direct evidence of interfacial interactions.

**Figure 3:**
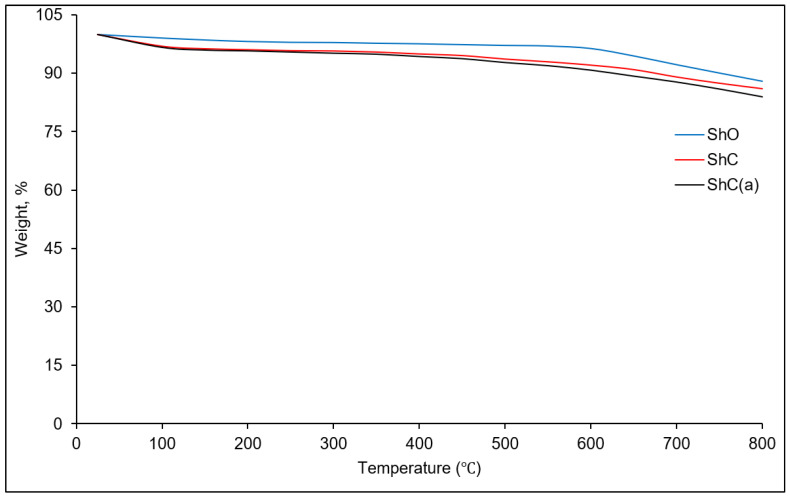
TG curves of the shungite fillers.

The effect of flotation enrichment and acid activation on the thermal stability of the shungite fillers was evaluated by thermogravimetric analysis (TGA) (Figure 3). It was shown that ShO loses no more than 3 wt% up to 600 °C, which is most likely associated with the loss of adsorbed and bound water. ShC and ShC(a) lose up to 3 wt% up to 100 °C. Up to 800 °C, ShC and ShC(a) lose an additional 5 wt% of the sample mass. This behavior presumably arises from the formation of hydroxides during flotation and activation, which decompose upon heating to form the corresponding oxides and water.

**Figure 4:**
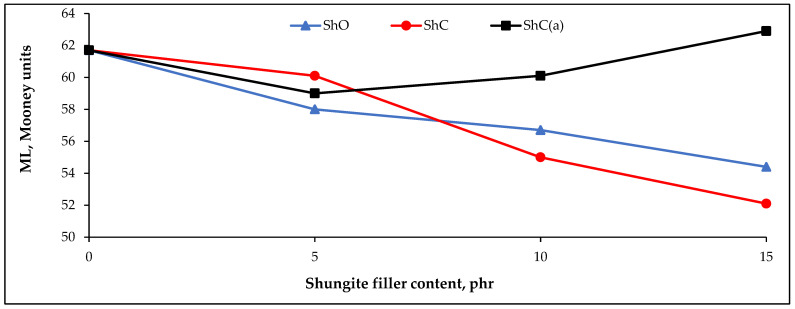
Effect of the shungite fillers on the Mooney viscosity (ML (1 + 4) 100 °C) of the rubber compounds.

An important characteristic that affects the uniform distribution of ingredients in the rubber compound and, consequently, the physicomechanical properties of the rubbers is their viscosity. According to the Mooney viscosity data, the viscosity of the rubber compounds is influenced by both the type and the amount of shungite filler. In particular, partial replacement of CB P 803 with ShO or ShC in the rubber formulations leads to a gradual decrease in Mooney viscosity with increasing shungite filler content (Figure 4).

Conversely, when more than 5 phr of the acid-activated concentrate ShC(a) is introduced, the Mooney viscosity begins to increase (Figure 4). Such behavior can be attributed to the high carbon content, the developed surface area (Table 3), and the large number of oxygen-containing groups on the surface of ShC(a), which enhance rubber–filler interactions and promote structuring, leading to reduced macromolecular mobility and, consequently, an increase in the structural viscosity of the compound [56,57].

**Figure 5:**
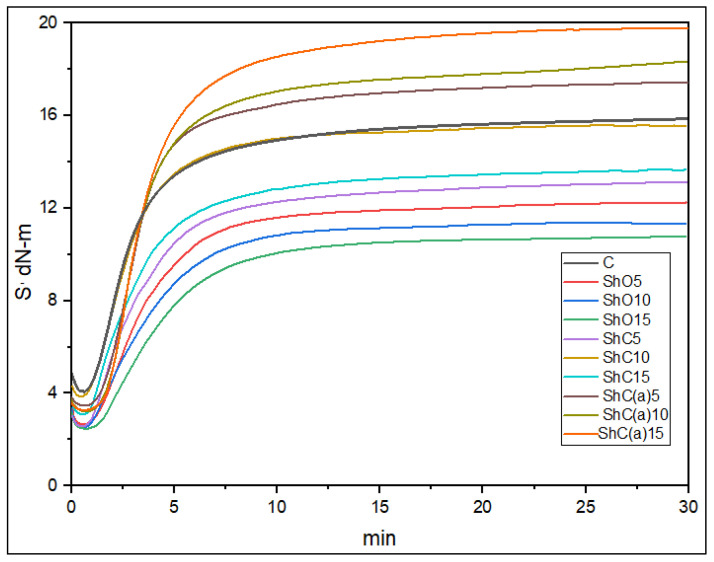
Rheometric vulcanization curves of the rubber compounds 160 °C, 30 min).

Analysis of the rheometric vulcanization curves of the rubber compounds at 160 °C showed that the use of the shungite fillers (Figure 5, Table 4) increases both the scorch time (t_s_) and the time to reach the optimum curing time (t_90_).

This behavior is presumably related to the fact that, upon the introduction of the shungite fillers, components of the vulcanization system are adsorbed on their surface and the metal oxides (Ca^+2^, K^+^) present in shungite compete with zinc oxide in the salt-forming reaction with stearic acid. As a result, the effective concentration of the active sulfur–accelerator complexes decrease, which slows down the formation of the three-dimensional crosslinked network [33,55].

The changes in the minimum torque (ML) values for the samples containing shungite fillers correlate with the Mooney viscosity values obtained for the rubber compounds under study. The decrease in the maximum torque upon the introduction of the initial shungite filler is associated with its low dispersity and weak reinforcing effect, together with the competition of the mineral phase with the sulfur–zinc curing system, which leads to a reduction in vulcanizate stiffness. In contrast, ShC(a) enriched in the carbonaceous phase and characterized by an increased specific surface area, enhances physicochemical interactions with the rubbers and promotes the formation of bound rubber, which is accompanied by an increase in vulcanizate stiffness and a rise in the maximum torque. The obtained result is consistent with a number of previous studies [58,59,60,61]. The vulcanization of the rubber compounds was carried out taking into account the rheometric data obtained.

The key requirements for oil- and fuel-resistant rubbers, which are widely used under exposure to aggressive media (petroleum products, oils, and solvents), are the retention of their physicomechanical properties and high thermal stability [62].

First, the properties of the rubbers were determined under normal conditions. It was shown that replacing CB P 803 with ShO leads to a decrease in the tensile strength of the rubbers (Table 5). Moreover, the greater the amount of CB replaced with ShO, the lower the tensile strength of the samples. This may be attributed to its low dispersity and the predominantly mineral, weakly organophilic nature of ShO. Replacement of CB with the carbon-enriched ShC, which contains a smaller fraction of the mineral phase and has a higher specific surface area, makes it possible to maintain the tensile strength at the level of the control sample. A similar effect is observed when CB is replaced with the acid-treated ShC(a); at the same loading, the tensile strength in this case further increases by 7–9% compared with the control sample.

It should be noted that the elongation at break increased compared with the control for all samples containing ShO and its derivatives ShC and ShC(a). Presumably, the introduction of the shungite fillers leads to a decrease in the crosslink density and stiffness of the vulcanizates due to their weak reinforcing effect and the partial retardation of the sulfur–zinc curing system. These findings are consistent with our previously conducted studies [33,55].

The largest changes in properties after thermo-oxidative aging for 72 h at 100 °C, relative to the initial values, are observed for the samples containing ShO. In the compositions with ShC, the magnitude of these changes is comparable to that of the control, whereas for the samples with ShC(a) the changes are smaller or at the level of the control (Table 5). This behavior is presumably related to the composition of the shungite fillers. ShO is characterized by a low carbon content (11 wt%) and a high fraction of the mineral phase, including metal oxides (SiO_2_, CaO, MnO, etc.) capable of catalyzing radical oxidative processes. In ShC, the carbon content increases to 39 wt% and the fraction of SiO_2_ and several metal oxides decreases, resulting in changes in properties after aging that are reduced to the level of the control rubber. In ShC(a), the carbon content is even higher (55 wt%) and several oxides (CaO, MnO, etc.) are practically removed, which further suppresses the catalytic decomposition of hydroperoxides and enhances the barrier role of the carbon phase, thereby ensuring minimal changes in the performance characteristics of the vulcanizates under thermo-oxidative aging (Table 5). Thus, ShC(a) provides the best stability of properties under elevated-temperature service conditions, whereas the use of ShO may lead to pronounced changes in these properties.

**Figure 6:**
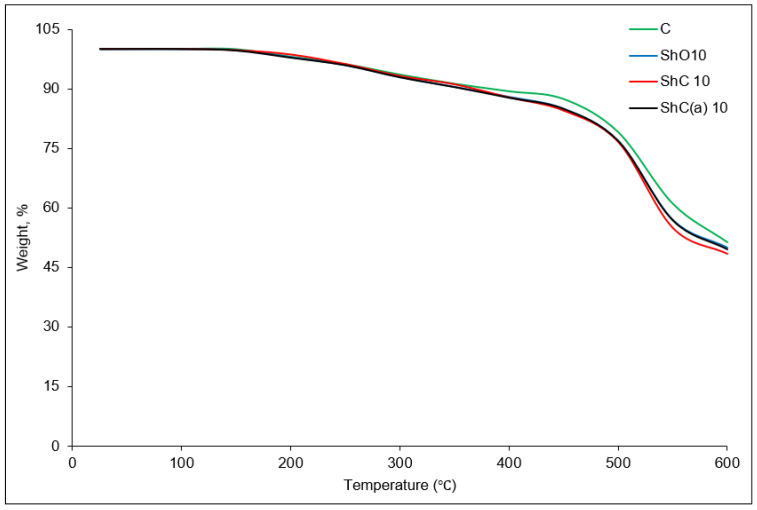
TG curves of the vulcanizates filled with the shungite fillers.

Analysis of the thermogravimetric curves of the vulcanizates (Figure 6) shows that the onset decomposition temperature is the same for all samples (200 °C), i.e., the introduction of the shungite fillers does not affect the formation of volatile products.

Thermogravimetric analysis showed that the incorporation of the shungite fillers affects the mass-loss rate of the rubber composites at elevated temperatures only to a minor extent. The main mass-loss region (200–450 °C) is associated with the degradation of processing additives and copolymer components of the rubber matrix, whereas the rapid mass-loss stage at higher temperatures is predominantly related to thermal decomposition of the main polymer backbone.

The presence of solid mineral shungite particles does not result in a significant additional release of volatile products or the appearance of new degradation pathways in this temperature range; therefore, their influence on the mass-loss rate during the stages of thermal degradation remains relatively weak. This behavior is consistent with the general characteristics of TGA of polymer composites, in which the matrix polymer dominates the main decomposition stages, while the effect of inert fillers on sample mass loss is typically limited.

The effect of the shungite fillers on the thermal stability of the rubbers in the initial mass-loss region is presented in Table 6, based on the temperatures corresponding to 5, 10, and 15% mass loss.

**Figure 7:**
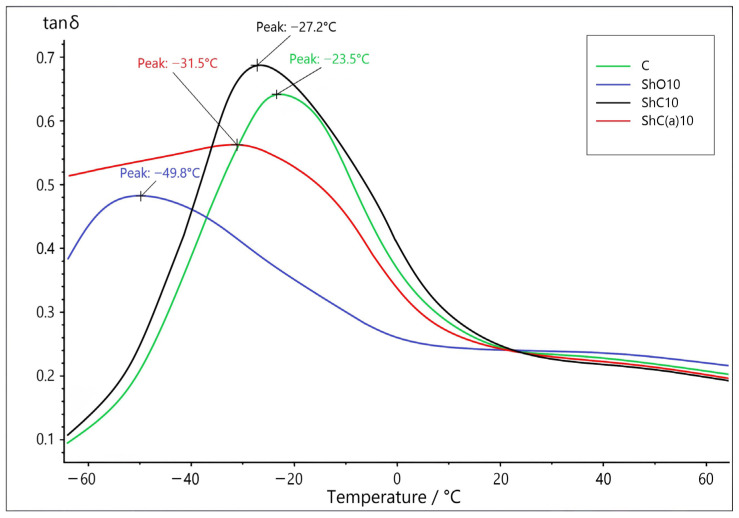
DMA curves of the vulcanizates filled with the shungite fillers.

Figure 7 presents the results of dynamic mechanical analysis (DMA) for the control compound and compositions containing various shungite fillers. The control compound without shungite fillers exhibits a maximum of the mechanical loss tangent (*T_g_*) at −23.5 °C. With the addition of shungite ore (ShO10), a pronounced decrease in *T_g_* to −49.8 °C is observed. In contrast, for samples containing shungite concentrate and acid-activated shungite concentrate, the shift in tan δ is less pronounced, occurring at −27.2 °C and −31.5 °C, respectively.

The reduction in the glass transition temperature can be attributed to the combined effect of the filler on the segmental dynamics of the rubber matrix. According to the literature, a significant decrease in *T_g_* in polymer composites is often associated with a plasticization effect caused by the migration of low-molecular-weight organic components from the filler into the matrix, which increases polymer chain mobility and lowers *T_g_* [63]. At the same time, the possible contribution of microstructural factors and specific interfacial interactions cannot be excluded, as they may also affect the temperature transitions in filled systems. Thus, the observed expansion of the low-temperature service range of the composites should be considered in light of these features.

An important performance characteristic of rubbers used for the manufacture of oil- and fuel-resistant pressure hoses is the stability of their properties in hydrocarbon media. Therefore, the changes in the strength properties of the rubbers after exposure in oil for 72 h were determined. The experiments showed that the introduction of ShO and its derivatives does not impair the oil resistance of the rubber. The change in tensile strength of the samples after exposure was small (Table 7) and did not exceed 2.7%. This indicates that partial replacement of CB with ShO and its enriched derivatives does not deteriorate the stability of the rubber properties in petroleum products for the formulations considered.

In evaluating the resistance of the rubbers to aggressive media, it was found that after exposure of samples containing various amounts of ShO, ShC and ShC(a) in oil for 72 h at 23 ± 1 °C, the mass change did not exceed +0.16%. On this basis, it can be concluded that ShO, ShC and ShC(a) do not impair the resistance of the rubbers to standard liquid for rubber SJR-1 (standard hydrocarbon medium for evaluating the properties of rubbers and rubber technical products).

## 4. Conclusions

In this work, shungite ore was modified by flotation followed by acid activation, and the effect of shungite ore and its carbon-enriched concentrates on the properties of oil- and fuel-resistant rubbers based on a blend of nitrile butadiene rubber and butadiene–α-methylstyrene rubber was investigated. Based on the experiments carried out, the following conclusions can be drawn. Flotation enrichment of shungite ore followed by acid activation of the concentrate increases the carbon content from 11% to 39% and 55%, and decreases the SiO_2_ content from 49% to 13% and 9%, respectively. At the same time, the specific surface area of the carbon-enriched filler increases by a factor of 2–2.5. Partial replacement of carbon black in the rubber formulations with shungite ore or flotation-enriched shungite concentrate leads to a gradual decrease in Mooney viscosity compared with the sample without shungite fillers. In contrast, the acid-activated shungite concentrate, at loadings above 5 phr, increases the viscosity of the rubber compound. This effect is attributed to the large number of oxygen-containing groups on its surface, which form additional interactions within the “rubber–filler” system. All shungite fillers increase both the scorch time and the optimum cure time, which is attributed to the presence of oxide impurities that compete in the vulcanization activation reactions. The acid-activated shungite concentrate provides high tensile strength and good thermo-oxidative stability of the rubbers, whereas the use of shungite ore at loadings above 5 phr reduces the tensile strength and may lead to a pronounced change in this parameter upon thermo-oxidative aging. It has been shown that the shungite fillers lower the glass transition temperature of the rubbers, thereby extending the low-temperature service range of the products. Upon immersion in oil, rubbers containing shungite ore and its carbon-enriched concentrates retain their mechanical properties; the best resistance in hydrocarbon media is exhibited by the rubber filled with the acid-activated shungite concentrate.

## Figures and Tables

**Table 1:** Rubber compound formulations with the shungite fillers.

Ingredients		Rubber Compound Designation									
		C *	ShO5	ShO10	ShO15	ShC5	ShC10	ShC15	ShC(a)5	ShC(a)10	ShC(a)15
		Content, phr (Parts per Hundred Rubber)									
1	BNKS-18 AMN	40	40	40	40	40	40	40	40	40	40
2	BNKS-28 AMN	50	50	50	50	50	50	50	50	50	50
3	SBR-1705 HI-AR	10	10	10	10	10	10	10	10	10	10
4	Technical sulfur	2	2	2	2	2	2	2	2	2	2
5	MBTS	1.5	1.5	1.5	1.5	1.5	1.5	1.5	1.5	1.5	1.5
6	ZnO	3	3	3	3	3	3	3	3	3	3
7	Stearic acid	1	1	1	1	1	1	1	1	1	1
8	IPPD	2	2	2	2	2	2	2	2	2	2
9	DBP	25	25	25	25	25	25	25	25	25	25
10	Petroleum resin	7	7	7	7	7	7	7	7	7	7
11	Petroleum bitumen	8	8	8	8	8	8	8	8	8	8
12	CB P 514	20	20	20	20	20	20	20	20	20	20
13	CB P 803	100	95	90	85	95	90	85	95	90	85
14	ShO	0	5	10	15	0	0	0	0	0	0
15	ShC	0	0	0	0	5	10	15	0	0	0
16	ShC(a)	0	0	0	0	0	0	0	5	10	15

* Control sample.

**Table 2:** Composition of oil from the Romashkinskoye oilfield.

Sulfur, %	Mechanical Impurities, %	Resin Content, %	Asphaltenes, %	Paraffin, %	Iron Sulfide, mg/L	Chloride Salts	
						mg/L	%
3.62	0.3	12.65	6.33	2.06	17.71	6023.35	0.66

**Table 3:** Characteristics of the shungite fillers.

Component	Content, wt%		
	ShO	ShC	ShC(a)
C	11.0 ± 0.5	39.0 ± 0.5	55 ± 0.5
Na_2_O	1.77 ± 0.1	1.39 ± 0.1	1.43 ± 0.1
MgO	1.31 ± 0.1	1.57 ± 0.1	0.81 ± 0.1
Al_2_O_3_	21.13 ± 0.1	20.26 ± 0.1	20.30 ± 0.1
SiO_2_	49.44 ± 0.1	13.64 ± 0.1	9.26 ± 0.1
P_2_O_5_	0.22 ± 0.01	<0.10 ± 0.01	<0.10 ± 0.01
K_2_O	3.42 ± 0.1	3.50 ± 0.1	4.73 ± 0.1
CaO	2.47 ± 0.1	1.16 ± 0.1	<0.10 ± 0.01
TiO_2_	3.06 ± 0.1	2.17 ± 0.1	2.36 ± 0.1
MnO	0.20 ± 0.01	<0.10 ± 0.01	<0.10 ± 0.01
Fe_2_O_3_	6.75 ± 0.1	9.37 ± 0.1	6.05 ± 0.1
Specific surface area, m^2^/g	9	17	23

**Table 4:** Parameters of the rheometric vulcanization curves of the rubber compounds (160 °C, 30 min).

Parameter	Rubber Compound Designation									
	C	ShO5	ShO10	ShO15	ShC5	ShC10	ShC15	ShC(a)5	ShC(a)10	ShC(a)15
ML, dN‧m	4.04	2.66	2.58	2.49	2.61	2.86	3.00	3.52	3.31	3.24
MH, dN‧m	15.61	12.28	11.48	10.83	14.18	13.93	13.69	17.52	18.35	19.93
∆M, dN‧m	11.57	9.62	8.80	8.34	11.57	10.70	10.69	14.00	15.04	16.69
t_10_, min	1.30	1.61	1.50	1.78	1.28	1.26	1.21	1.79	1.99	1.95
t_90_, min	6.86	8.16	8.34	9.78	8.97	9.73	8.70	8.05	8.93	8.88
R_v_ = 100/(t_90_ − t_10_)	17.98	15.26	14.62	12.50	13.00	11.08	13.35	15.97	14.40	14.43

**Table 5:** Physicomechanical properties of the rubbers.

Parameter *	Sample Designation									
	C	ShO5	ShO10	ShO15	ShC5	ShC10	ShC15	ShC(a)5	ShC(a)10	ShC(a)15
	Initial physicomechanical properties of the rubbers									
TS, MPa	9.7	8.5	7.4	7.1	8.9	10.1	9.4	9.8	10.0	10.1
ε, %	293	306	327	327	363	360	363	303	306	333
ε_res_, %	4.0	12.0	9.3	9.3	8.0	8.0	6.7	1.3	2.7	2.7
R, %	28	32	32	33	28	28	28	28	28	28
HSA	65	67	63	62	66	67	66	68	68	68
Physicomechanical properties of the rubbers after thermal aging (72 h at 100 °C)										
TS, MPa	11.1	10.4	9.9	10.1	10.1	10.7	10.3	10.2	10.3	10.0
ε, %	210	236	250	274	253	233	240	210	203	220
R, %	18	21	21	20	20	20	20	19	18	19
HSA	71	74	71	71	73	75	73	76	75	75
** Relative change in the physicomechanical properties of the rubbers after thermal aging (72 h at 100 °C)										
∆ fp, %	+14.43	+22.35	+33.78	+42.25	+13.48	+5.94	+9.57	+4.08	+3.0	−1.0
∆ ε, %	−28.33	−22.88	−23.55	−16.21	−30.30	−35.28	−33.88	−30.69	−33.66	−33.93
∆ R, %	−34.70	−34.27	−34.75	−38.18	−28.98	−28.62	−28.21	−33.99	−34.06	−30.80
∆ HSA, %	+11.52	+10.55	+12.64	+14.05	+10.98	+10.70	+10.82	+11.32	+9.66	+9.52

* TS—tensile strength; ε—elongation at break; ε_res_—residual elongation, R—rebound resilience; HSA—Shore A hardness. ** The relative change in the parameter was calculated using the following equation: Δ*x* = $\frac{\left(x-x_{0}\right)}{x_{0}}\times 100,\;\%\;$, where *x*_0_ is the initial value and *x* is the current value after thermal aging.

**Table 6:** Mass loss of the vulcanizates in thermogravimetric analysis.

Sample	Mass Loss, %			
	5	10	15	20
	Temperature (°C) at Mass Loss			
Rubber without shungite (C)	270	360	470	494
ShO10	250	325	445	485
ShC10	262	350	445	480
ShC(a)10	270	360	450	480

**Table 7:** Physicomechanical properties of the rubbers after exposure in oil for 72 h.

Parameter *	Samples									
	C	ShO5	ShO10	ShO15	ShC5	ShC10	ShC15	ShC(a)5	ShC(a)10	ShC(a)15
TS, MPa	10.2	8.5	7.6	7.2	9.0	10.4	9.5	9.8	10.2	10.2
∆ TS, %	+5.2	0.0	+2.7	+1.4	+1.1	0.0	+1.0	0.0	+2.0	+1.0
∆m, %	+0.14	+0.11	+0.13	+0.15	+0.12	+0.14	+0.16	+0.13	+0.14	+0.15

* TS—tensile strength.

## Data Availability

The raw data supporting the conclusions of this article will be made available by the authors on request.

## Abbreviations

ShO	Shungite ore
ShC	Shungite concentrate
ShC(a)	Shungite concentrate acid-activated
TGA	Thermogravimetric Analysis
DMA	Dynamic Mechanical Analysis
